# Managing experts’ conflicts of interest in the EU Joint Clinical Assessment

**DOI:** 10.1136/bmjopen-2024-091777

**Published:** 2024-11-18

**Authors:** Arianna Gentilini, Iva Parvanova

**Affiliations:** 1 The London School of Economics and Political Science, London, UK; 2 Department of Economics and Public Policy, Imperial College London, London, UK; 3 Department of Political Science, LUISS Guido Carli, Roma, Italy

**Keywords:** Health policy, ETHICS (see Medical Ethics), THERAPEUTICS

## Abstract

**Abstract:**

**Objective:**

This article critically evaluates the European Commission’s 2024 Implementing Regulation (IR) on conflicts of interest (COIs) management for stakeholders in the European Union (EU) Joint Clinical Assessment (JCA), with a focus on individual experts such as clinicians and patient representatives.

**Key findings:**

The IR is the first EU-level framework to assess COIs in the context of health technology assessment (HTA). The regulation requires experts involved in the JCA to submit annual declarations of interest for both financial and non-financial interests and presents a matrix on whether these conflicts should disqualify them from participating in the joint work. We compared the IR to COIs-management approaches from other European national HTA bodies and found that the IR is closely modelled after the French guidelines. Concerns include potential over-representation of experts from a small number of countries, lack of guidance on organisational COIs, and ambiguities in how the size of financial interests are disclosed. Unclear resource allocation for enforcement could also hinder compliance.

**Conclusions:**

The IR marks progress in EU-wide HTA collaboration, but improvements in transparency, expert diversity, and comprehensive COIs management are needed to ensure impartiality in the JCA process.

## Background

 In 2021, the European Union (EU) adopted a regulation for joint clinical assessment (JCA) of new health technologies.^1^ The JCA aims to speed up approval and reduce duplication of work among EU Member States by providing a high-quality review of the clinical evidence submitted by the manufacturer for use of national health technology assessment (HTA) bodies.^1^ The regulation will apply to oncology drugs and advanced therapy medicinal products starting in January 2025, and by 2030, it will encompass all new health technologies.^2^

Including stakeholders, such as physicians and patients, in the assessment of health technologies is vital for fully capturing the value of medicinal products and devices.^3^ However, concerns over conflicts of interest (COIs) of individual experts and members of stakeholder organisations have raised questions about the impartiality of the process.^4^,^6^ The involvement of conflicted experts erodes trust in public institutions and can delay access to medicines.^7^ This was highlighted earlier this year in a ruling by the Court of Justice of the EU, which stated that the decision by the European Medicines Agency to deny marketing authorisation to Hopveus, a medicine for treating alcohol dependency, should be reverted in light of the involvement of an expert who was connected with one of the sponsor’s competitors.^8^

In October 2024 the European Commission (EC) adopted an Implementing Regulation (IR) on COI management among stakeholders involved in the JCA. ^9^ This builds on a draft Implementing Act (IA) published in May 2024 and the feedback from a public consultation where interested parties could express their perspectives on the draft. ^10^ In light of the adoption of these novel regulatory tools, we aim to critically appraise the new COI management framework in the IR by focusing on what it means for individual experts, such as clinicians and members of the patient community.

## Managing COIs in the JCA

The IR requires individual experts involved in JCA to submit their curriculum vitae and signed declarations of interests (DOIs), the latter of which must be updated annually or whenever a change occurs. The EC will assess DOIs to identify COIs, requesting additional information if needed. Identified conflicts may result in exclusion or limited participation in joint activities. Submitted DOIs and CVs will be made publicly available online to ensure transparency, except for patients' data for privacy reasons. The IR also includes a matrix to determine whether a stakeholder can participate in the joint activities given their declared interests. Importantly, the IR takes into account both financial and other types of interests relevant to the work which experts are due to the participate in .^11^ Stakeholders are required to declare conflicts in the following seven categories, both currently and within the past three to five years: employment, consultancy, strategic advisory roles, financial interests of the expert or their immediate family members, involvement as principal investigator and lead membership in an organisation funded by a health technology developer (HTD).

**Figure 1:**
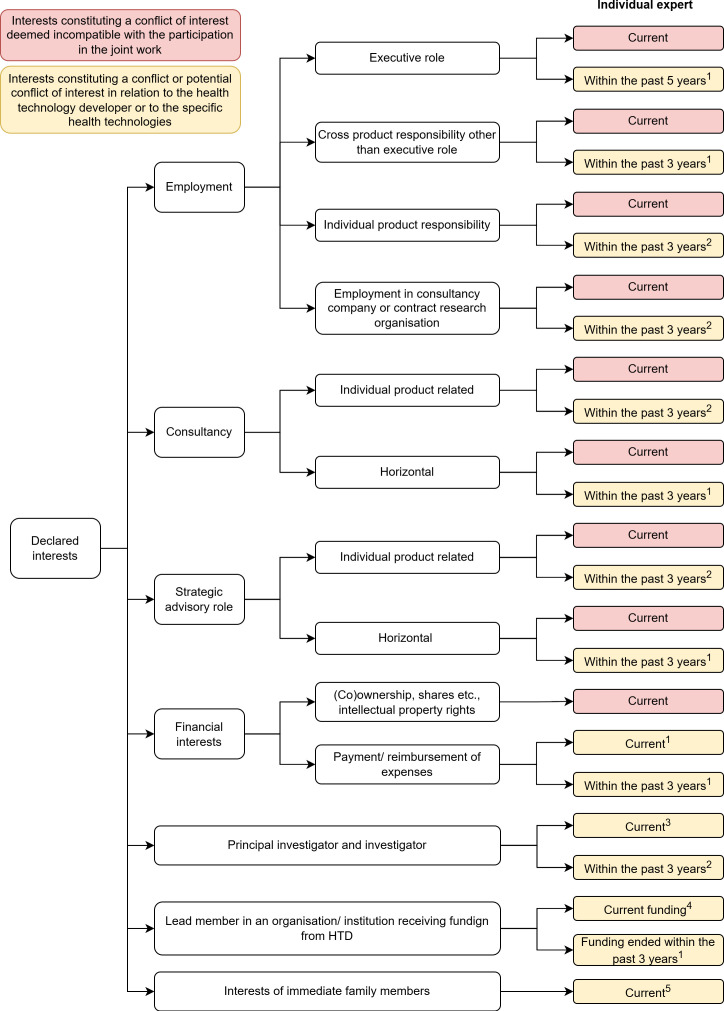
COIs decision matrix for individual experts. Abbreviations: HTD, health technology developer. ^1^No involvement of the individual expert in joint work in relation to health technologies under assessment or comparator health technologies from the HTD, where the role, horizontal responsibility, presentation, training course, participation in a conference/seminar/event is/was related to a therapeutic area or several therapeutic areas, or if the therapeutic area cannot be identified, in relation to any health technologies from the HTD. ^2^No involvement of the individual expert in joint work in relation to specific health technologies under assessment or comparator health technologies, defined as a health technology identified in the assessment scope for the joint clinical assessment. ^3^If the clinical trial, observation study, clinical investigation or performance analysis is/was sponsored by an HTD, no involvement of the individual expert in joint work in relation to any health technologies from the HTD. If the trial or study is/was sponsored by other means except by HTDs, no involvement of the individual expert in joint work in relation to specific health technologies under assessment or comparator health technologies. ^4^No involvement of the individual expert in joint work in relation to specific health technologies under assessment or comparator health technologies from the health technology developer. ^5^In the case of intellectual property rights, no involvement of the individual expert in joint work in relation to specific health technologies under assessment or comparator health technologies. In all other instances, no involvement of the individual expert in joint work in relation to any health technologies from the HTD.

In figure 1, we illustrate how COIs might limit experts’ participation in joint work according to this matrix. Depending on the time and category of conflict, experts might be deemed unsuitable to participate in the assessment of a specific or any technology by this developer, a competitor or in the relevant therapeutic area.

Experts with current employment, consultancy, advisory roles or stocks, or those holding stocks, shares, or intellectual property rights with an HTD are disqualified from participating in joint work due to conflicts of interest. Financial interests, whether direct or indirect, have varied repercussions. For example, receiving expense reimbursements from a HTD prohibits the expert from involvement in joint work related to products from the same HTD. However, the expert might be allowed to participate in joint work on a different therapeutic area if the conflict is limited to a specific disease. Similarly, experts serving as principal investigators in clinical trials funded by an HTD cannot participate in joint work related to any of that HTD’s products. If the expert oversees an institution receiving funding from the HTD, they are also barred from participating in joint work related to any of the HTD’s technologies. Additionally, conflicts involving family members may disqualify experts from joint activities related to a specific HTD or entirely, depending on the nature of the declared interests.

On the other hand, past conflicts have more nuanced consequences. Experts who held executive roles or had horizontal responsibilities within an HTD are disqualified from participating in joint work related to any of the manufacturer’s technologies within the therapeutic area if one can be identified, and overall, otherwise. However, previous involvement with a specific technology or a third-party contractor only excludes the expert from assessing that particular technology. For example, if an expert previously served as the Chief Scientific Officer at Company X and was responsible for overseeing the development of breast cancer technologies, they would be barred from participating in joint assessments of any of the company's drugs within the therapeutic area, if one can be identified, and overall, otherwise. In contrast, if the same expert's involvement with Company X was limited to serving as a consultant specifically for the development of a single breast cancer drug, they would only be disqualified from participating in assessments related to that particular drug, while remaining eligible to assess other products from the company outside this particular health technology or its comparators. Similarly, past consultancy or advisory roles limit the expert’s involvement in the same way depending on whether they were involved in an individual product or had horizontal responsibilities. Past financial interests are treated similarly to current ones. For example, receiving expense reimbursements from an HTD prohibits the expert from involvement in joint work related to products from the HTD in the relevant therapeutic area if one can be identified, and overall, otherwise. Experts who previously led institutions funded by an HTD are disqualified from participating in joint work related to the HTD’s products. Finally, those who served as principal investigators in trials funded by the HTD, cannot take part in the joint work linked to the specific health technologies under assessment or comparator health technologies.

### Situating the IA draft within the COIs regulatory context in Europe

The new EU regulation does not explicitly refer to existing guidelines from other HTA bodies in Europe. However, to assess how it compares to existing COIs frameworks, we reviewed the approaches adopted by a selected number of HTA bodies, namely, the French Haute Autorité de Santé (HAS), the UK National Institute for Health and Care Excellence (NICE), and the German Gemeinsamer Bundesausschuss (G-BA).^12^,^14^

The IR shares some similarities with the reviewed national guidelines. First, all define COIs as a situation where personal interests may influence or appear to influence an expert’s impartiality and objectivity, and they require experts involved in HTA work to report COIs through standardised declarations. HAS requires a Public Declaration of Interests; G-BA requires a Self-Declaration of Potential Conflicts of Interest; NICE requires declarations at appointment and regular updates. Additionally, all bodies state that their assessment is not universal but rather takes a case-by-case approach, leading to the exclusion of experts when their conflicts is deemed to significantly compromise impartiality according to the evaluating committees.

On the other hand, there are some differences between the EU JCA COIs management approach and the other HTA bodies’ guidelines, especially regarding what payments need to be reported. For example, HAS and G-BA require any financial payment above €250 to be declared, while NICE requires all relevant financial interests to be declared regardless of the amount. This differs from the current approach taken by the draft legislation, where payments and reimbursements of a value below €1,000 from a HTD within the past three years need not be declared. Overall, our review suggests that the IR is closely modelled after the French approach. Most notably, they both have share similar categories of interests in their declarations (eg, paid or unpaid employment over the previous 5 years). However, the French guidelines discuss how the HAS committee evaluating COIs will be able to triangulate data between the experts’ DOIs and the Transparence Santé database. This is a freely accessible repository where stakeholders such as physicians, hospitals, and patient and professional organisations must report the financial payments received from the pharmaceutical industry.^15^ The EU JCA lacks such mechanism, which could increase the accountability of the experts’ submitted declarations and provide the evaluating committee with a tool to validate statements.

## Assessing the COIs management matrix

^15^ The perspectives of stakeholders from these countries might not fully reflect the challenges and experiences of all EU members, ultimately limiting the relevance and applicability of their input to the clinical practices in different jurisdictions. Additionally, unequal representation might be problematic in the case of rare diseases, where their low prevalence translates to few if any non-conflicted patient and clinical experts, due to close involvement with industry-led initiatives.^16^

While the IR is an important first step towards the management of COIs in the appraisal of new technologies at the EU-level, there are aspects that need to be clarified.

First, there is a risk of overrepresentation from countries with well-established HTA systems that routinely involve experts, such as France and Germany. ^16^ The perspectives of stakeholders from these countries might not fully reflect the challenges and experiences of all European Union members, ultimately limiting the relevance and applicability of their input to the clinical practices in different jurisdictions. Additionally, unequal representation might be problematic in the case of rare diseases, where their low prevalence translates to few if any non-conflicted patient and clinical experts, due to close involvement with industry-led initiatives.^16^

While the regulation acknowledges that conflicted experts should be included in the joint work if non-conflicted ones are unavailable^2^ ^9^(please see Article 7, recital 3) it remains unclear what mitigation strategies the committees will adopt to ensure that involving conflicted experts does not bias the outcome of the joint work. To limit enforcement challenges the IR should clarify how this process will deviate from the COI management matrix described above. Furthermore, the IR does not explain the recruitment process of stakeholders involved in the joint work, emphasising the importance of individuals’ expertise. Nevertheless, a selection process that ensures diversity of experts can mitigate the concerns discussed above. A potential solution is creating an online platform for the systematic collection of contributions from clinical and patient experts ^4^ . This approach would ensure participation from a wider range of experts across Member States, address concerns about overrepresentation, reduce the risk of industry sponsorship bias, and lessen the administrative burden for participants.

Second, experts are asked to complete their DOIs in an individual capacity. However, this overlooks the fact that patient and clinical experts often affiliated with to organisations which might have different conflicts. While it is important that individuals report their personal interests, they should also be required to disclose the financial interests of the organisations they belong to. The IR requires DOIs from experts who serve as lead members of organisations (eg, (co)chair, president, director, treasurer). We suggest that the DOI adds a section to collect organisational information, from all relevant individual experts regardless of their role in the organisation funded by the HTD or its competitors.

Third, some aspects of the IR are vague and might lead to implementation challenges. For example, it remains unclear whether experts should disclose the exact amount of financial payments they receive, indicate a range, or simply state if the sum exceeds the specified threshold of €1,000 cumulatively over three years, without revealing by how much. Currently, the DOI form presented in the Appendix of the IR does not include a clear space where experts can indicate the description of and the monetary value associated with each declared conflict where this might be relevant, such as consultancy fees and stocks.^2^ Moreover, while some disqualifying conflicts, such as holding an executive role within an HTD are considered within the past 5 years, others are only limited to 3. The EC needs to be more transparent in explaining how these rules have been formulated to minimise potential appeals from disqualified stakeholders or HTDs. Similarly, the IR outlines many HTA Secretariat responsibilities, such as ensuring compliance with the process, reviewing DOIs, and assessing COIs, but does not specify the resources (both personnel and financial) allocated to ensure enforcement.^2^To address these, we suggest that the EC ensures full clarity and transparency across the recruitment, selection, and exclusion processes to reduce the potential of appeals and delays in the joint work.

## Conclusion

The JCA is an important step forward for collaboration in HTA processes across the EU, and holds the potential to reduce duplication of work, reduce access disparities among EU countries, and ensure timely patient access to health technologies. The approach to COIs management put forward in this IR will be an example across different jurisdictions. Addressing our concerns will help to ensure impartiality throughout the joint activities.

### Patient and public involvement

Due to the nature of the analysis, which focused on reviewing a proposed EU regulation on managing experts’ COIs, patients and the public were not involved in the design, conduct or reporting of this study.
