# Supplementary material for: Managing experts’ conflicts of interest in the EU Joint Clinical Assessment
Source: BMJ Open. 2024 Nov 18;14(11):e091777. doi: 10.1136/bmjopen-2024-091777 (PMC11574398; doi:10.1136/bmjopen-2024-091777)
Supplement: Supplementary file 1 [file bmjopen-2024-091777.reviewer_comments.pdf]

PEER REVIEW HISTORY

BMJ Open publishes all reviews undertaken for accepted manuscripts. Reviewers are asked to complete a checklist review form and are provided with free text boxes to elaborate on their assessment. These free text comments are reproduced below.

ARTICLE DETAILS

Title (Provisional)

Managing Experts' Conflicts of Interest in the EU Joint Clinical Assessment

Authors

Gentilini, Arianna; Parvanova, Iva

VERSION 1 - AUTHOR RESPONSE

Dear Florence,

We are delighted to hear that our article aligns with the scope of the communication piece and has piqued your interest. We are pleased to submit it for your review.

Please feel free to reach out if you have any questions.

Best wishes,

Arianna Gentilini

VERSION 2 - REVIEW

**Reviewer**	**1**
**Name**	**Lexchin, Joel**
**Affiliation**	**York University, School of Health Policy & Management**
**Date**	**31-Aug-2024**

COI **Between 2021-2024, Joel Lexchin received payments for writing a brief on the role of promotion in generating prescriptions for a legal firm, for being on a panel about pharmacare and for co-writing an article for a peer-reviewed medical journal. He is a member of the Boards of Canadian Doctors for Medicare and the Canadian Health Coalition. He receives royalties from University of Toronto Press and James Lorimer & Co. Ltd. for books he has written.**

This commentary discusses the newly published Implementation Act on COI management among stakeholders involved in joint clinical assessment of new health technologies. As someone who has written extensively about COI I welcome the European legislation but I think that the authors could do more to examine what is included and not included in the legislation and also how it originated and whether or not its effects are going to be evaluated.

1. What kind of consultation went into developing the new legislation? Were COI rules from other jurisdictions taken into account?

2. How does the new IA compare with legislation in other jurisdictions?

3. Are there plans to evaluate the effectiveness of the legislation with respect to minimizing COI?

4. The authors should provide information about whether declarations just cover financial COI, what level of payment constitutes a COI, whether the declarations cover future relationships with commercial entities and whether experts have to declare the exact amount of payments or just ranges.

5. Page 3, line 40: What is the basis for saying the IA is a robust first draft?

Minor points

1. Page 2, line 11: Spell out ATMP.

2. The authors need to name the journal in reference 7.

Reviewer 2

Name Chen, Yaolong

Affiliation Lanzhou University, Evidence-Based Medicine Centre, School of Basic Medical Sciences

Date 11-Sep-2024

COI No competing interests.

This article provides a clear analysis of the Implementing Act on conflicts of interest published by the European Commission. It outlines the specific situations in which experts would be disqualified from participating and highlights areas where the Implementing Act (IA) still requires improvement. The suggestions provided are valuable for the future enhancement of the IA. However, before the manuscript is accepted, it is suggested to refine some details.

Minor Comments:

1. Please review the 'Keywords' section on the cover page. It is unclear if the '<' symbol between 'Health policy' and 'HEALTH SERVICES ADMINISTRATION & MANAGEMENT' holds any specific meaning.

2. The keywords listed on the cover page are not consistent with those mentioned on Page 1, Line 36. It is recommended to verify and ensure consistency.

3. It is advisable to provide the full form of the term 'CV' on Page 2, Line 35.

VERSION 2 - AUTHOR RESPONSE

Reviewer: 1
Dr. Joel Lexchin, York University
Comments to the Author:
This commentary discusses the newly published Implementation Act on COI management among stakeholders involved in joint clinical assessment of new health technologies. As someone who has written extensively about COI I welcome the European legislation but I think that the authors could do more to examine what is included and not included in the legislation and also how it originated and whether or not its effects are going to be evaluated.

1. What kind of consultation went into developing the new legislation? Were COI rules from other jurisdictions taken into account?
Response: There is no public information about any initial consultation which went into designing the version of the Implementing Act we critique in this manuscript. The public consultation carried out by European Commission (EC) took place between 29 May 2024 - 26 June 2024. The aim was to gather stakeholders' perspectives on the current draft. 81 contributions were published online on the website of the EC including interventions from pharmaceutical and medical device companies, business associations, non-governmental and patient organizations and academic institutions. Those varied in their degree of support. The EC has not commented on whether and to what extent they will address the contributions to this public consultation. The IA is planned for adoption by the end of 2024. We discuss how COI rules were considered below.
We have included some information about the public consultation and adoption timeline in text.

In May 2024, The European Commission (EC) published an Implementing Act (IA) on COI management among stakeholders involved in the JCA and carried out a public consultation where interested parties could express their perspectives on the draft.[9] In light of the upcoming adoption of the regulation by the end of 2024, this article aims to critically appraise the IA by focusing on what it means for individual experts, such as clinicians and members of the patient community.

2. How does the new IA compare with legislation in other jurisdictions?

Response: Many thanks for your comment, which we feel has significantly improve the quality and breadth of the piece. We have taken this opportunity to review the COI management approach of other HTA agencies, namely NICE, HAS and G-BA to provide further context to the EU legislation. The changes are tracked in the document and are also reported below.

1. ***Situating the IA draft within the COI regulatory context in Europe***

The new EU regulation does not explicitly refer to existing guidelines from other HTA bodies in Europe. However, to assess how it compares to existing COI frameworks, we reviewed the approaches adopted by a selected number of HTA bodies, namely, the French Haute Autorité de Santé (HAS), the UK National Institute for Health and Care Excellence (NICE), and the German Gemeinsamer Bundesausschuss (G-BA).[11-13]

All the reviewed guidelines share similarities. First, all define a conflict of interest as a situation where personal interests may influence or appear to influence an expert's impartiality and objectivity, and they require experts involved in HTA work to report COIs through standardised declarations. HAS requires a Public Declaration of Interests; G-BA requires a Self-Declaration of Potential Conflicts of Interest; NICE requires declarations at appointment and regular updates. Additionally, all bodies state that their assessment is not universal but rather takes a case-by-case approach, leading to the exclusion of experts when their conflicts can significantly compromise impartiality according to the evaluating committees.

the EU JCA COI management approach and the other HTA bodies' guidelines, especially regarding what payments need to be reported. For example, HAS and G-BA require any financial payment above 250 EUR to be declared, while NICE requires all relevant financial interests to be declared regardless of the amount. This differs from the current approach taken by the draft legislation, where payments and reimbursements of a value below 1,000 EUR from a HTD within the past three years need not be declared. Our review suggests that the IA is closely modelled after the French approach. For example, both have similar categories of interests in their declarations (e.g., paid or unpaid employment over the previous 5 years). A notable difference between the two, is that the French guidelines discuss how the HAS committee evaluating COIs will be able to triangulate data between the experts' declarations of interest and the Transparence Santé database, a freely accessible repository where stakeholders such as physicians, hospitals, and patient and professional organisations must report the financial payments received from the pharmaceutical industry. [14] *This mechanism increases the accountability of the experts' submitted declarations and provides the evaluating committee with a tool to validate statements.*

3. Are there plans to evaluate the effectiveness of the legislation with respect to minimizing COI?

Response: Thank you for the question. After an extensive search, we could not find any indication that the European Commission will carry out an evaluation of the effectiveness of the legislation is planned. While we agree that this would be an important next step, discussing such an evaluation would fall beyond the scope of this analysis which focuses on the design and implementation of the IA.

4. The authors should provide information about whether declarations just cover financial COI, what level of payment constitutes a COI, whether the declarations cover future relationships with commercial entities and whether experts have to declare the exact amount of payments or just ranges.

Response: Stakeholders are required to declare interests in the following four categories, both currently and within the past three to five years: employment, consultancy, strategic advisory roles, and financial interests. Interests of immediate family members are also taken into account. Figure 1 describes types of interests within these 4 main categories. Within the consultancy and strategic advisory role categories, experts need to also consider whether they might provide advice/express opinions on the strategy, direction or development activities of a health technology developer and declare this. Importantly, the declaration of interest form which is published as an Appendix to IA does not include any space where experts can actually indicate the exact amount or range of the relevant interest. We have now included a discussion of this in our piece (see Section 4).

Most importantly, it remains unclear whether experts should disclose the exact amount of financial payments they receive, indicate a range, or simply state if the sum exceeds the specified threshold, without revealing by how much. Currently, the DOI form presented in the Appendix of the IA does not include any space where experts can indicate the monetary value associated with the declared conflict where this might be relevant, such as consultancy fees and stocks.

We have also now noted explicitly that the COI management framework covers both financial and other interests relevant to the work experts are expected to participate in.

Importantly, the IA takes into account both financial and other types of interests relevant to the work which experts are due to the participate in.[10]

5. Page 3, line 40: What is the basis for saying the IA is a robust first draft?

Response: Thank you for your comment. We agree with the suggestion and have therefore revised the wording as follows:

While the IA is an important first step towards the management of COIs in the appraisal of new technologies at the EU-level, there are aspects that need to be clarified.

Minor points

1. Page 2, line 11: Spell out ATMP.

Response: Thank you, we have now spelled out ATMP (Advanced Therapy Medicinal Products) in the updated draft.

2. The authors need to name the journal in reference 7.

Response: Many thanks, we have now updated the reference to include the name of the journal.

Reviewer: 2
Prof. Yaolong Chen, Lanzhou University
Comments to the Author:
This article provides a clear analysis of the Implementing Act on conflicts of interest published by the European Commission. It outlines the specific situations in which experts would be disqualified from participating and highlights areas where the Implementing Act (IA) still requires improvement. The suggestions provided are valuable for the future enhancement of the IA. However, before the manuscript is accepted, it is suggested to refine some details.

Minor Comments:
1. Please review the 'Keywords' section on the cover page. It is unclear if the '<' symbol between 'Health policy' and 'HEALTH SERVICES ADMINISTRATION & MANAGEMENT' holds any specific meaning.
Response: Many thanks for your comment. Unfortunately, we are unable to amend this as is created automatically by the submission system.
2. The keywords listed on the cover page are not consistent with those mentioned on Page 1, Line 36. It is recommended to verify and ensure consistency.
Response: As explained above, the choice of keywords in the submission platform are limited so we cannot ensure they are perfectly aligned.
3. It is advisable to provide the full form of the term 'CV' on Page 2, Line 35.
Response: We have changed it as suggested in “curricula vitae”.
